# Prevalence and factors associated with road traffic crashes among truck drivers in Southeast Iran

**DOI:** 10.1371/journal.pone.0320974

**Published:** 2025-04-09

**Authors:** Raheleh Hashemi Habybabady, Hassan Okati-Aliabad, Mohammad Sabouri, Mahdi Mohammadi, Alireza Ansari-Moghaddam

**Affiliations:** 1 Health Promotion Research Center, Zahedan University of Medical Sciences, Zahedan, Iran; 2 Student Research Committee, Zahedan University of Medical Sciences, Zahedan, Iran; Shahrood University of Medical Sciences, IRAN, ISLAMIC REPUBLIC OF

## Abstract

Road traffic injuries are the second leading cause of death in Iran. The study investigated the prevalence and the influencing factors of Road traffic crashes (RTCs) among truck drivers in southeast Iran. In this cross-sectional study, 592 truck drivers were recruited using a multi-stage sampling method from November 2022 to February 2023. Data was collected through a researcher-administered questionnaire that included the crashes, individual characteristics, driving characteristics, work patterns, sleep and fatigue-related factors, workload, driving styles, and personality traits. Simple and multiple logistic regressions were used to assess the association between risk factors and crash involvement in the 3 last years. The surveyed drivers had a mean age of 37.4 ± 8.9 years, with an average driving history of 13.7 ±  7.6 years. Among the respondents, 28.4% reported involvement in crashes over the last 3 years, with 12.1% reporting one, 10% reporting two, and 6.3% experiencing three or more crashes. A significant portion of the crashes (42.5%) occurred between midnight and 6:00 a.m. In their lifetime, 24.2% of participants reported at least one sleep-related crash, 40.5% reported at least one fatigue-related crash, and 6.9% reported at least one crash resulting in a fatality. The odds of RTCs were higher among truck drivers who used drugs (OR = 2.03, 95% CI, 1.36-3.04), used mobile devices for texting (OR = 2.88, 95% CI, 1.56-5.30), neglected seat belt usage (OR = 1.81, 95% CI, 1.10-2.99), had accumulated traffic fines in the last year (OR =  8.18, 95% CI, 3.82–17.52, OR =  11.39, 95% CI, 5.42–23.92, OR =  17.78, 95% CI, 7.50–42.17, for 1-2, 3-6, and > 6 traffic fines, respectively), consumed sleeping pills (OR = 2.52, 95% CI, 1.19-5.35), engaged sleep driving (OR = 11.30, 95% CI, 7.18-17.80), extended their driving hours without a break (OR =  3.02, 95% CI, 1.55–5.87, for consecutive driving hours before taking a break ≥ 8), experienced fatigue while driving (OR =  1.98, 95% CI, 1.24–3.17, for sometimes experienced fatigue while driving), faced high visual demands (OR = 1.23, 95% CI, 1.02-1.50), exhibited a careless driving style (OR = 0.95, 95% CI, 0.91- 0.99), and had higher levels of neuroticism (OR = 1.05, 95% CI, 1.01-1.10). The study sheds light on the significant prevalence of road traffic crashes among truck drivers. The findings underscore a constellation of factors amplifying crash risks within this occupational group. These outcomes emphasize the multifaceted nature of road safety issues within the trucking industry, indicating the need for targeted interventions and preventive measures to enhance driver safety and reduce the incidence of road traffic crashes among truck drivers.

## Introduction

Road traffic injuries are the eighth leading cause of death globally and represent the primary cause of mortality among individuals aged 15 to 29 years [1]. The fact that by 2030 road traffic injuries will be the seventh cause of death in the world is growing [2]. In 2017, there were 1,243,068 fatalities related to road injuries out of a total of 54,192,330 new cases of road injuries worldwide [3]. Road injuries are anticipated to impose an economic burden of US$1.8 trillion on the world economy from 2015 to 2030 [4]. In Iran, road traffic injuries are the second leading cause of death [5]. There has been a decline in road traffic injuries’ incidence, prevalence, mortality, and associated disabilities over the past two decades in Iran. Among the provinces of Iran, Sistan and Baluchistan had the highest age-standardized disability-adjusted life-years (DALY) rates [6].

Road Traffic Crashes (RTCs) are influenced by various factors such as traffic conditions, vehicle-related factors, and most significantly, human-related factors, which play a central role in contributing to RTCs [7]. Evidence shows that over a third of all crashes are caused by human errors [8].

In the context of road safety, heavy vehicles have a critical role to play. Evidence reveals that as the proportion of heavy vehicles increases in each lane, average travel time also increases. Moreover, lanes with more heavy vehicles experience more frequent lane-changing maneuvers by passenger cars. This effect is amplified at higher traffic densities and when heavy vehicles make up a larger percentage of the traffic. An increase in the percentage of heavy vehicles to 30% is associated with a 5% higher likelihood of crashes, which could potentially reduce overall traffic safety [9]. In addition, the analysis of crash rates shows that the proportion of commercial vehicles on roads has a significant impact on crash rates. This impact varies depending on the type of vehicle, crash severity, and road characteristics [10].

Unsafe driving behaviors are the primary cause of truck crashes [11]. The evidence revealed that 40% of truck drivers exhibited markedly dangerous driving tendencies [12]. Truck drivers often have to drive under adverse conditions like fatigue, bad weather, or traffic due to delivery constraints [13]. Investigation of fatal road crashes showed that drivers impaired by drugs or alcohol exhibited a higher incidence of risky behaviors like speeding, failure to use a seatbelt, and driving without a valid license. These risk factors were prevalent among the majority of drug/alcohol-impaired fatally injured drivers and more than half of the sober drivers involved in fatal crashes [14].

In recent years, there has been a significant shift in the factors contributing to crashes, with driver-related elements like distractions being implicated in a substantial proportion of crashes. Distractions, particularly from handheld electronic devices, pose a substantial risk to driver safety due to their widespread use [15]. Notably, cell phone use increases the risk of a culpable crash by 70% [16]. Among long-haul truck drivers, frequent mobile phone users are 29 times more likely to be involved in a crash than non-frequent users [17]. Data analysis from a comprehensive naturalistic driving study revealed that texting and calling on cell phones while driving are widespread practices. Moreover, individuals who engage in texting at higher rates tend to have an increased risk of being involved in crashes [18]. This heightened risk can be attributed to the fact that texting while driving is a distracting activity that significantly impairs driving performance. It compromises drivers’ ability to focus and divide their attention effectively, thereby increasing the risk of life-threatening traffic events [19].

A meta-analysis indicated a significant association between being involved in crashes and driving while fatigued [20]. Furthermore, fatigue ranks among the primary contributing factors resulting in fatal crashes, particularly when attributed to the large truck driver’s responsibility [21]. Investigating truck crashes showed that around 10.8% of drivers had multiple physiological risk factors for fatigue at the time of the crash [22].

Evaluating crash risk using detailed naturalistic driving data indicated that alcohol and drug impairment significantly raises the likelihood of being involved in a crash or near-crash event by 34% [23]. Alcohol or drug impairment was a frequent contributing factor in fatal RTCs, with a higher prevalence when the motor vehicle driver was killed compared to cases where the driver survived [24].

Evidence has shown a positive association between crash involvement and drowsy driving [20]. In truck drivers, sleepiness affects various safety-related performance aspects, with sleep duration showing a stronger association with accidents and accident risk compared to sleep quality [25]. Overall, sleep apnea and insufficient sleep (less than 7 hours) were found to contribute to 10% and 9% of motor vehicle crashes, respectively [26].

The evidence indicated that factors such as extended driving duration, the number of working days per week, rest patterns, inadequate sleep hours, and a history of violations were significantly linked to drowsy driving among long-haul truck drivers [27]. Extended periods of driving, resulting in fatigue and sleepiness, played a significant role in causing crashes involving heavy vehicles [28]. In truck drivers, the sleep pattern linked to the highest safety-critical event rate involves shorter sleep duration, sleeping during the early stage of a non-work period, and reduced sleep between 1 a.m. and 5 a.m. [29]. A survey among professional drivers in Korea revealed that working over 12 hours a day and experiencing excessive daytime sleepiness were linked to consistently engaging in risky driving behaviors [30].

The lack of regular breaks was correlated with a higher risk of crashes in heavy vehicle drivers [31]. Increasing the total rest-break duration and taking more rest breaks can consistently reduce fatigue-related crash risk, with two rest breaks typically sufficient for a 10-hour trip. Shorter rest breaks of around 30 minutes are usually adequate. However, taking rest breaks too soon after starting a trip may reduce their effectiveness [32]. Analyzing the impact of driving hours and rest breaks on truck driver safety revealed that the statistical significance of crash odds ratios is only observed for the 11th driving hour. Taking 1, 2, or 3 rest breaks can decrease crash odds by 68%, 83%, and 85%, respectively, compared to drivers who did not take any breaks [33].

There is evidence that mental workload has a significant impact on road safety [34]. Increased cognitive load can affect gaze behavior and driving performance [35] and eye movements and cognitive workload play a role in lateral position variability [36].

Different driving styles play a moderating role in the relationship between job strain and occupational traffic crashes among professional drivers [37]. Driving styles characterized by maintaining lower speeds and exhibiting adaptive responses to driving conditions are associated with fewer crashes in the occurrence of safety-critical events [38]. Aggressive drivers tend to disregard the state of the car behind them in the target lane, increasing the risk of lane-changing crashes [39].

Personality traits exerted indirect effects on crash risk by influencing risky driving behaviors, although they showed no direct impact on crash risk [40]. A recent systematic review and meta-analysis revealed that risky and aggressive driving behaviors were negatively associated with conscientiousness, agreeableness, and openness, although they were positively associated with neuroticism [41].

Understanding RTCs and associated factors among truck drivers needs a comprehensive study. With human-related factors identified as pivotal contributors to crashes, this study aimed to investigate the relationship between crash risk with the unique challenges faced by truck drivers, including fatigue, drug use, cell phone distraction, and long hours of driving. Additionally, this study sought to explore the relationship between crash risk and mental workload, driving styles, and personality traits among truck drivers.

## Materials and methods

### Study area

This study was conducted in three regions within the Sistan and Baluchistan province, situated in the southeast of Iran. Selected for being the second-largest province in Iran and its proximity to Afghanistan and Pakistan, Sistan and Baluchistan face distinctive transportation challenges with primarily two-way roads featuring a single narrow lane on each side. Region I (Zahedan): The provincial capital, Zahedan, serves as a crucial regional center near the Afghanistan and Pakistan borders, playing a vital role in international trade connections and economic activities.

Region II (Mirjaveh): Mirjaveh, a border city in the east, functions as a key road crossing to Pakistan, fostering trade and travel links between the two countries.

Region III (Milk): The village of Milk, positioned on the Afghanistan-Iran border, acts as a strategic link facilitating trade and communication. The Milk-Zaranj border crossing connects landlocked Afghanistan to international waters through Iran.

**Fig 1:**
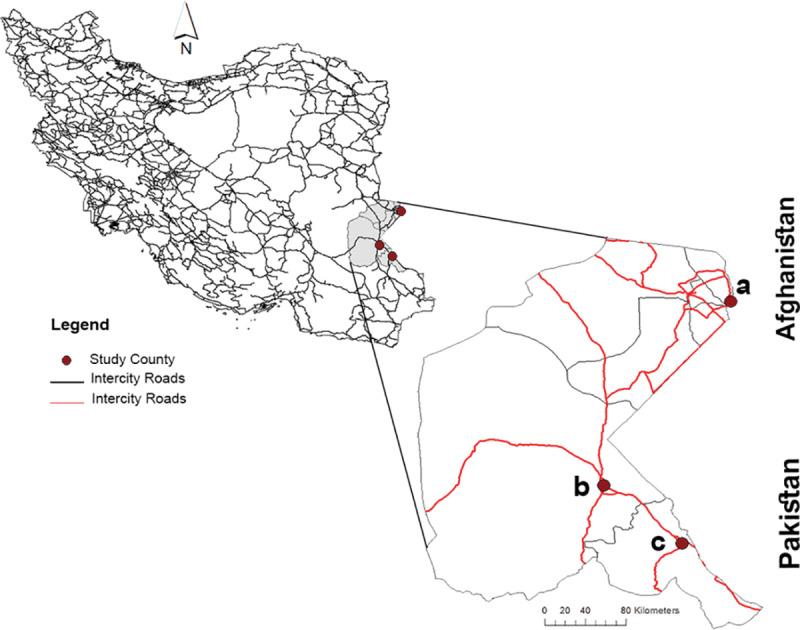
Map of Iran and the study area. Three regions highlighted in the Iran map indicate the study area. The map depicted in this figure was created using ArcGIS version 10.4 (Esri, Redlands, CA, USA).

These study regions are depicted in Figure 1, showcasing the geographical distribution and significance of the research locations.

### Study design and participants

We conducted a cross-sectional survey among 592 truck drivers in Sistan and Baluchistan province. The participants were selected through a multi-stage sampling method. In the first stage, through purposive sampling and in consultation with traffic police experts as well as road and transportation experts, different groups of truck drivers and places where drivers had the most traffic were identified. These groups included Iranian and non-Iranian drivers who mostly used the routes leading to Zahedan as the capital of the province where all the transportation routes to the north and south of the province end. Also, Iranian and non-Iranian drivers crossing the Milak border in the northern part of the province were identified, as traveling to Afghanistan. Similarly, drivers were identified at the Mirjaveh border city in the eastern part of the province, traveling to Pakistan. In the second stage, the gathering places of truck drivers who are responsible for moving cargo in the province and to and from Afghanistan and Pakistan were identified. These places were gasoline pump stations located at the entrances and exits of Zahedan and the border terminal of Milak and Mirjaveh. In the last stage, in each of the mentioned points, people were selected proportional to size and by a simple random sampling method. In this way, one out of every 3 truck drivers entering the gasoline pump stations or terminals was randomly selected. Drivers who were fluent in Farsi, at least 18 years old, had at least one year of driving experience, and operated trucks with a weight exceeding 6 tons were eligible to participate in the study.

### Data collection

Data was collected from November 2022 to February 2023. The data collection was conducted during both morning and evening hours in Zahedan, specifically at gasoline pump stations, the Milak border, and the border city of Mirjaveh locations where drivers were required to stop. The participants completed a researcher-administered questionnaire that included crash history in the last 3 years, individual characteristics, driving characteristics, work patterns, sleep and fatigue-related factors, workload, driving styles, and personality traits.

Individual characteristics included nationality, age, education, marital status, driving experience, smoking, drug use, alcohol use, cell phone type, type of phone use, and aim of phone use while driving. Driving characteristics included type of driving license, car owning, car tonnage, gearbox type, using a seatbelt while driving, and number of driving fines in the last year.

To explore drivers’ fatigue experiences, we inquired about the onset time of fatigue while driving, the duration of consecutive driving hours before taking a break, the average break duration per stop, and the frequency of experiencing fatigue while driving.

The Global Satisfaction with Sleep (GSD) was employed to assess sleep satisfaction. Individuals were categorized as experiencing GSD if they indicated their sleep satisfaction as rather unsatisfactory, quite unsatisfactory, or completely unsatisfactory [42]. Additionally, participants reported a history of eating something to stay awake while driving, taking sleeping pills while driving, napping while driving, experiencing sleep driving, and having a driving schedule timetable.

Additionally, participants shared information on their average daily driving hours, the number of crashes in the past three years, past 12 months, past 6 months, as well as their lifetime crash history due to fatigue and sleepiness and lifetime fatal crash history. Furthermore, they reported the time of the accidents.

We employed the Driving Activity Load Index (DALI) for subjectively assessing driving workload. DALI is a modified version of the NASA-TLX, tailored to the demands of the driving task. DALI evaluated the effort of attention, visual demand, auditory demand, situational stress, temporal demand, and interference on a scale ranging from 0 to 5 [43].

The study assessed participants’ personality traits using The Big Five Inventory–2-Short Form (BFI-2-S) a concise tool with 30 items evaluating five major personality domains including extraversion, agreeableness, conscientiousness, neuroticism, and openness [44]. Participants rated their agreement with statements on a scale from 1 to 5.

The multidimensional driving style inventory was used to investigate the driving style of truck drivers [45]. This inventory contains 44 items and evaluates 8 driving styles including chaotic, anxious, dangerous, violent, impulsive, relaxed, calm, and careful. Respondents indicated the degree of fit of each item with their feelings, thoughts, and behavior while driving in a 6-point range from not at all to very much.

### Data analysis

The data were analyzed using the Statistical Package for Social Sciences (SPSS) version 19. Qualitative variables were presented using frequency distribution, while mean±SD described quantitative variables. The comparison between Iranian and non-Iranian drivers utilized the chi-square test. Factors associated with crashes in the last 3 years were identified through simple and multiple logistic regressions. For the odds ratio (OR), a 95% confidence interval (CI) was presented. The significance level for the study was set at 0.05.

### Ethical considerations

The study obtained ethical approval from Zahedan University of Medical Sciences (IR.ZAUMS.REC.1401.074), and informed consent was secured from participants, ensuring voluntary participation, confidentiality, and data security through the use of serial numbers for participant identification.

## Results

In this study, 592 truck drivers were surveyed, with an average age of 37.4 ± 8.9 and an average driving history of 13.7 ± 7.6 years. A majority of the drivers were married (83.6%) and held a secondary or high school degree (73.1%). Notably, 30.6% reported smoking, 24.7% acknowledged drug use, and 9% admitted to alcohol consumption. Furthermore, a significant portion of drivers used smartphones (75%), primarily for making calls (90.4%), and a majority used hand-held cell phones (65.7%) (Table 1).

**Table 1:** Association between individual characteristics and RTCs in the last 3 years.

	Total	Crash in the last 3 years	One-factor Model		Multi-factor Model	
	N (%)	N (%)	OR (95% CI)	P_value	OR (95% CI)	P_value
Nationality				**0.004**		
Iranian		112 (25.3)	1.00			
non-Iranian		55 (37.7)	1.79(1.20,2.66)			
Age (year)				**0.689**		
≤30	170 (28.7)	47 (27.6)	1.00			
31-40	228 (38.5)	68 (30.2)	1.13(0.73,1.76)	0.577		
41-50	133 (22.5)	33 (24.8)	0.86(0.52,1.45)	0.579		
>50	61 (10.3)	19 (31.1)	1.18(0.63,2.24)	0.604		
Education				**0.063**		
Illiterate	41 (6.9)	15 (36.6)	1.00			
Elementary school	57 (9.6)	15 (26.3)	0.62(0.26,1.47)	0.278		
Secondary school	176 (29.7)	54 (30.9)	0.77(0.38,1.58)	0.480		
High school	257 (43.4)	59 (23.1)	0.52(0.26,1.05)	0.068		
University graduate	61 (10.3)	24 (39.3)	1.12(0.50,2.55)	0.779		
Marital status				**0.021**		
Single	73 (12.4)	22 (30.1)	1.00			
Married	494 (83.6)	132 (26.9)	0.85(0.50,1.46)	0.561		
Divorced	24 (4.1)	13 (54.2)	2.74(1.06,7.06)	0.037		
Driving experience (year)				**0.187**		
≤5	78 (13.2)	18 (23.1)	1.00			
6-15	319 (53.9)	94 (29.7)	1.41(0.79,2.51)	0.250		
16-25	141 (23.8)	45 (32.1)	1.58(0.84,2.98)	0.159		
>25	54 (9.1)	10 (18.5)	0.76(0.32,1.80)	0.529		
Smoking				**0.739**		
Yes	181 (30.6)	53 (29.3)	1.07(0.73,1.57)			
No	411 (69.4)	114 (27.9)	1.00			
Drug use				**<0.001**		0.001
Yes	146 (24.7)	58 (39.7)	2.02(1.36,3.00)		2.03(1.36,3.04)	
No	446 (75.3)	109 (24.6)	1.00		1.00	
Alcohol use				**0.206**		
Yes	53 (9.0)	19 (35.8)	1.46(0.81,2.65)			
No	539 (91.0)	148 (27.6)	1.00			
Cell phone type				**0.533**		
Smart	444 (75.0)	128 (29.0)	1.14(0.75,1.74)			
Non-smart	148 (25.0)	39 (26.4)	1.00			
Type of phone use				**0.148**		
Hand-held	389 (65.7)	105 (27.2)	1.00			
Hands-free	119 (20.1)	41 (34.5)	1.41(0.91,2.18)	0.128		
Bluetooth headset	49 (8.3)	9 (18.4)	0.60(0.28,1.28)	0.189		
Vehicle system	35 (5.9)	12 (34.3)	1.40(0.67,2.91)	0.372		
Aim of phone use while driving				**0.002**		0.003
Making/ answering phone calls	535 (90.4)	140 (26.3)	1.00		1.00	
Sending/Reading messages	47 (7.9)	24 (51.1)	2.92(1.60,5.34)	<0.001	2.88(1.56,5.30)	0.001
Using the Internet and social networks	10 (1.7)	3 (30.0)	1.20(0.31,4.71)	0.794	1.34(0.34,5.32)	0.674

**Fig 2:**
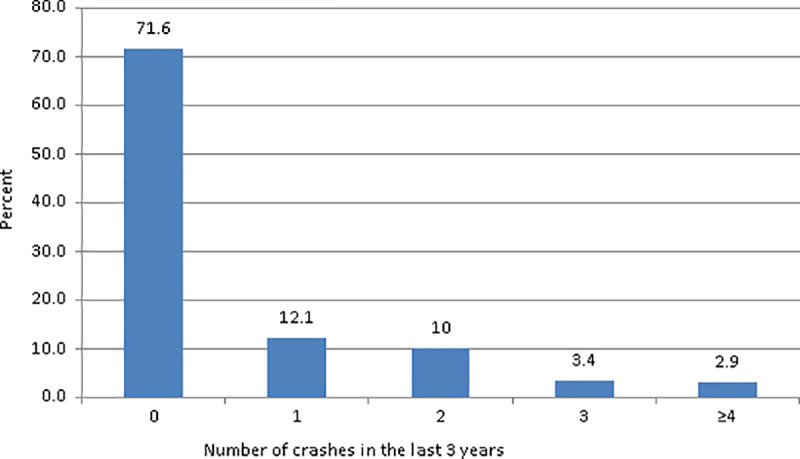
Frequency distribution of the number of crashes in the last 3 years.

**Fig 3:**
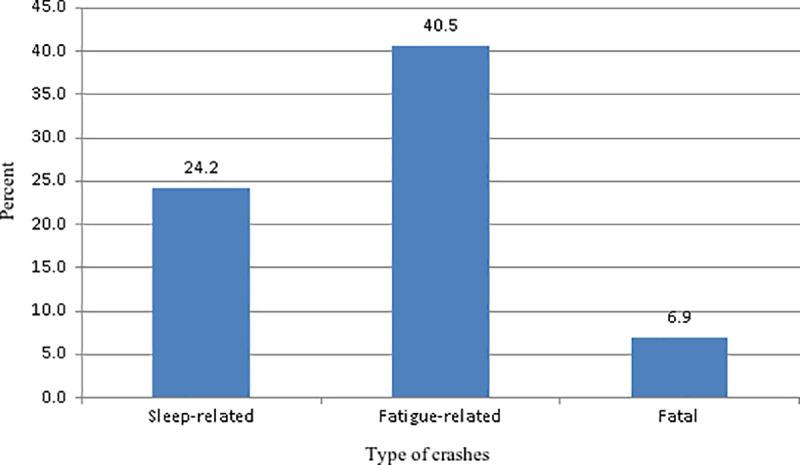
Lifetime history of sleep-related crashes, fatigue-related crashes, and crashes resulting in fatality.

Among the drivers surveyed, 28.4% reported involvement in crashes over the last 3 years, with 12.1% reporting one, 10% reporting two, and 6.3% experiencing three or more crashes (Figure 2). Additionally, there were 12.7% of crashes within the last 6 months. Most of the crashes (42.5%) happened between midnight and 6:00 a.m. In their lifetime, 24.2% of the participants reported experiencing at least one sleep-related crash, 40.5% reported at least one fatigue-related crash, and 6.9% reported at least one crash resulting in a fatality (Figure 3).

**Fig 4:**
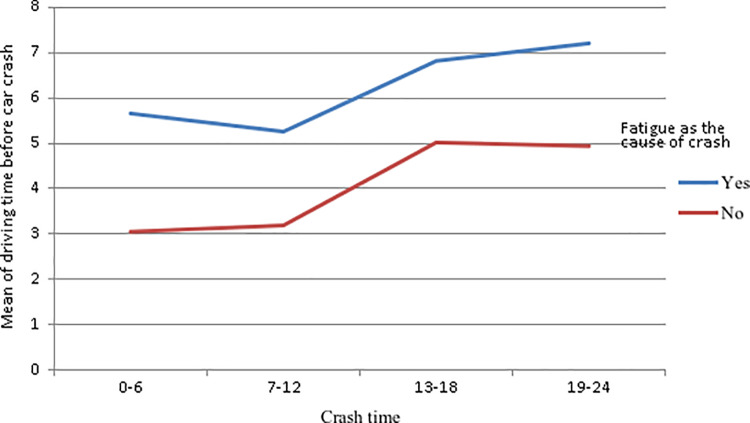
Mean of driving time before the crash according to the time of crash and fatigue as the cause of the crash.

Figure 4 illustrates the mean of driving time before the crash for fatigue-related and non-fatigue-related crashes at different crash times. The mean driving time before the crash in fatigue-related crashes was 6.03 ± 3.55 compared to 4.10 ±  2.54 in non-fatigue-related crashes (P < 0.001). 83% of crashes occurring between midnight and 6:00 a.m. were fatigue-related, compared to 61.5% of crashes between 7:00 a.m. and midnight (OR = 3.04, 95% CI: 1.78, 5.22, P < 0.001).

In a one-factor model, the odds of experiencing a crash in the last 3 years were significantly higher among Iranian drivers (OR = 1.79, P = 0.004), divorced individuals compared to those who were single (OR = 2.74, P = 0.037), drug users (OR = 2.02, P < 0.001), and drivers engaged in sending/reading messages compared to those making phone calls (OR = 2.92, P < 0.001). Moreover, in the multifactor model, both drug use (OR = 2.03, P = 0.001) and the practice of sending/reading messages compared to making/answering phone calls (OR = 2.88, P = 0.001) remained significantly associated with crash risk (Table 1).

A majority of drivers did not possess the vehicle (56.3%), held a basic 1 driving license (66.7%), adhered to seatbelt usage while driving (81.1%), and had at least one driving fine within the past year (72.4%). Additionally, a significant proportion of trucks featured a manual transmission system (89.2%), with half of them falling into the medium or heavy tonnage category (Table 2). According to a multi-factor model, the odds of experiencing a crash in the last 3 years increased due to the absence of seatbelt use (OR = 1.81, P = 0.022) and the number of driving fines (P < 0.001) (Table 2).

**Table 2:** Association between driving characteristics and RTCs in the last 3 years.

	Total	Crash in the last 3 years	One-factor Model		Multi-factor Model	
	N (%)	N (%)	OR (95% CI)	P_value	OR (95% CI)	P_value
Type of driving license				**0.335**		
Basic 1	392 (66.7)	106 (27.1)	1.00			
Basic 2 & 3	196 (33.3)	60 (30.9)	1.20(0.82,1.76)			
Car owning				**0.939**		
Driver	333 (56.3)	94 (28.2)	1.00			
Owner	259 (43.8)	73 (28.5)	1.01(0.71,1.46)			
Car tonnage				**0.371**		
Light truck (>10000)	293 (49.6)	76 (26.2)	1.00			
Medium truck (10001-20000)	59 (10.0)	15 (25.4)	0.96(0.51,1.82)	0.901		
Heavy truck (20001-45000)	239 (40.4)	75 (31.4)	1.29(0.88,1.88)	0.190		
Gearbox type				**0.156**		
Automatic	64 (10.8)	23 (35.9)	1.48(0.86,2.56)			
Manual	528 (89.2)	144 (27.4)	1.00			
Using a seatbelt while driving				**0.093**		0.022
Yes	480 (81.1)	128 (26.8)	1.00		1.00	
No	112 (18.9)	39 (34.8)	1.46(0.94,2.26)		1.81(1.10,2.99)	
Number of driving fines in the last year				**<0.001**		<0.001
0	158 (27.6)	9 (5.7)	1.00		1.00	
1-2	167 (29.1)	52 (31.5)	7.57(3.58,16.00)	<0.001	8.18(3.82,17.52)	<0.001
3-6	192 (33.5)	75 (39.1)	10.54(5.07,21.93)	<0.001	11.39(5.42,23.92)	<0.001
>6	56 (9.8)	28 (50.0)	16.44(7.01,38.58)	<0.001	17.78(7.50,42.17)	<0.001

Approximately half of the drivers were accustomed to eating while driving and adhering to scheduled work hours. A notable (7.8%) reported the use of sleeping pills, while 10.8% rated their sleep quality as poor. Strangely, incidents of napping while driving (20.4%) and sleep driving (24.2%) were reported. The majority of drivers worked more than 8 hours per day (67.9%) and experienced fatigue up to approximately 6 hours of driving (80.6%). Although 37.5% of drivers acknowledged fatigue setting in after 4 hours of driving, only 25.7% opted to take a break during that timeframe. The average rest time per stop was up to one hour for 91.6% of drivers and 58.5% of drivers reported driving while fatigued (Table 3).

**Table 3:** Association between work patterns, sleep, and fatigue-related factors and RTCs in the last 3 years.

	Total	Crashes in the last 3 years	One-factor Model		Multi-factor Model	
	N (%)	N (%)	OR (95% CI)	P_value	OR (95% CI)	P_value
Eating something to stay awake						
Yes	289 (48.8)	88 (30.8)	1.26(0.88,1.80)	0.207		
No	303 (51.2)	79 (26.1)	1.00			
Taking sleeping pill						0.016
Yes	46 (7.8)	23 (50.0)	2.77(1.51,5.09)	0.001	2.52(1.19,5.35)	
No	546 (92.2)	144 (26.5)	1.00		1.00	
Napping while driving						
Yes	121 (20.4)	63 (52.1)	3.80(2.50,5.77)	<0.001		
No	471 (79.6)	104 (22.2)	1.00			
Experiencing sleep driving						<0.001
Yes	143 (24.2)	96 (68.1)	11.33(7.33,17.51)	<0.001	11.30(7.18,17.80)	
No	449 (75.8)	71 (15.8)	1.00		1.00	
Having a scheduled timetable						
Yes	273 (46.1)	81 (29.8)	1.14(0.80,1.63)	0.477		
No	319 (53.9)	86 (27.1)	1.00			
Driver’s assessment of sleep quality				0.089		
Satisfied	527 (89.2)	143 (27.3)	1.00			
Dissatisfied	64 (10.8)	24 (37.5)	1.59(0.93,2.74)			
Daily driving hours				0.500		
≤8	190 (32.1)	54 (28.9)	1.00			
9-12	245 (41.4)	74 (30.2)	1.07(0.70,1.62)	0.765		
>12	157 (26.5)	39 (24.8)	0.81(0.50,1.32)	0.402		
Hours of driving causing driver fatigue				0.826		
≤4	222 (37.5)	61 (27.7)	1.00			
5-6	255 (43.1)	71 (27.8)	1.01(0.67,1.50)	0.978		
>6	115 (19.4)	35 (30.7)	1.16(0.70,1.90)	0.569		
Consecutive driving hours before taking a break				0.014		0.002
≤4	152 (25.7)	37 (24.7)	1.00		1.00	
5-7	352 (59.5)	94 (26.7)	1.11(0.72,1.73)	0.634	1.22(0.72,2.06)	0.463
≥8	88 (14.9)	36 (41.4)	2.16(1.22,3.80)	0.008	3.02(1.55,5.87)	0.001
Average break duration per stop (hour)						
≤1	542 (91.6)	150 (27.8)	1.00			
>1	50 (8.4)	17 (34.0)	1.34(0.72,2.47)	0.356		
Driver fatigue while driving				0.006		0.016
Newer and rarely	245 (41.5)	53 (21.7)	1.00		1.00	
Sometimes	301 (50.9)	96 (32.1)	1.70(1.16,2.52)	0.007	1.98(1.24,3.17)	0.004
Usually, most of the time and always	45 (7.6)	18 (40.0)	2.40(1.23,4.69)	0.010	1.89(0.82,4.34)	0.135

In a one-factor model, the odds of experiencing a crash in the last 3 years significantly increased with the use of sleeping pills (OR = 2.77, P = 0.001), napping while driving (OR = 3.80, P < 0.001), sleep-driving (OR = 11.33, P < 0.001), driving for eight or more hours compared to four or fewer hours (OR = 2.16, P = 0.008), and experiencing fatigue while driving (sometimes: OR = 1.70, P = 0.007, usually: OR = 2.40, P = 0.010). In a multifactorial model, the odds of a crash were elevated by the use of sleeping pills (OR = 2.52, P = 0.016), sleep-driving (OR = 11.30, P < 0.001), taking rest breaks for eight or more hours compared to four or fewer (OR = 3.02, P = 0.001), and experiencing fatigue while driving (sometimes: OR = 1.98, P = 0.004) (Table 3).

Among the subscales related to workload, the highest mean scores were observed for visual demand, effort of attention, and auditory demand, ranging from 80% to 65% of the maximum possible score, respectively. Conversely, the lowest mean score was associated with situational stress, amounting to approximately 25% of the maximum possible score. Notably, there was a significant correlation between visual demand and truck crashes (P = 0.033). Specifically, a one-unit increase in visual demand was found to elevate the odds of a crash by 23% (Table 4).

**Table 4:** Association between workload and RTCs in the last 3 years.

Crashes	Crashes		One-factor Model		Multi-factor Model	
	No	Yes	OR (95% CI)	P_value	OR (95% CI)	P_value
**Workload**	Mean ± SD	Mean ± SD				
Effort of attention	3.56 ± 1.17	3.74 ± 1.07	1.15 (0.98,1.36)	0.096		
Visual demand	3.97 ± 1.09	4.17 ± 0.79	1.23(1.02,1.50)	0.033	1.23(1.02,1.50)	0.033
Auditory demand	3.22 ± 1.06	3.39 ± 1.03	1.67(0.98, 1.39)	0.087		
Situational stress	1.17 ± 1.09	1.34 ± 1.26	1.13 (0.97,1.33)	0.116		
Temporal demand	3.01 ± 1.24	2.96 ± 1.26	0.97(0.84,1.12)	0.67		
Interference	2.92 ± 1.28	2.91 ± 1.32	0.99(0.86,1.15)	0.97		

Among the various driving style subscales, the highest mean scores were associated with driving styles characterized by care, patience, and reduced distress, ranging between 84% and 74% of the maximum possible score. In contrast, the mean scores for all other driving style subscales fell within the range of 35% to 45% of the maximum possible score. Within a one-factor model, being prone to anger emerged as a significant risk factor, while exhibiting careful driving tendencies was a noteworthy protective factor against truck crashes. Specifically, a one-unit increase in anger was linked to a 4% increase in crash odds (P = 0.025), whereas a one-unit increase in careful driving was associated with a 5% decrease in crash odds (P = 0.012). In the multi-factor model, the most pivotal driving style factor was identified as being careful (P = 0.012) (Table 5).

**Table 5:** Association between driving style and RTCs in the last 3 years.

	Crashes		One-factor Model		Multi-factor Model	
	No	Yes	OR (95% CI)	P_value	OR (95% CI)	P_value
**Driving style subscales**	Mean ± SD	Mean ± SD				
Dissociative	16.16 ± 5.59	17.10 ± 5.64	1.03(0.99,1.06)	0.075		
Anxious	16.29 ± 5.46	16.89 ± 5.05	1.02(0.99,1.06)	0.230		
Risky	10.44 ± 3.49	10.78 ± 3.74	1.03(0.98,1.08)	0.307		
Angry	13.12 ± 5.04	14.16 ± 4.56	1.04(1.01,1.08)	0.025		
High velocity	15.87 ± 5.61	16.50 ± 5.94	1.02(0.99,1.05)	0.242		
Distress reduction	17.46 ± 3.68	18.05 ± 3.62	1.05(0.99,1.10)	0.085		
Patient	19.14 ± 3.81	19.02 ± 3.90	0.99(0.94,1.04)	0.757		
Careful	25.28 ± 4.13	24.28 ± 4.30	0.95(0.91,0.99)	0.012	0.95(0.91,0.99)	0.012

Within the personality traits subscales, the highest mean scores were observed for agreeableness and conscientiousness, with participants scoring approximately 81% of the maximum possible score. In contrast, the lowest mean score was associated with the neuroticism trait, accounting for about 48% of the maximum possible score. In a one-factor model, agreeableness emerged as a significant protective factor, while neuroticism was identified as a risk factor for truck crashes. Specifically, a one-unit increase in agreeableness was linked to a 5% decrease in crash odds (P = 0.040), whereas a one-unit increase in neuroticism scores resulted in a 5% increase in crash odds (P = 0.027). In the multi-factor model, the most crucial factor was identified as neuroticism (P = 0.027) (Table 6).

**Table 6:** Association between personality traits and RTCs in the last 3 years.

	Crashes		One-factor Model		Multi-factor Model	
	No	Yes	OR (95% CI)	P_value	OR (95% CI)	P_value
**Personal traits**	Mean ± SD	Mean ± SD				
Extraversion	21.56 ± 3.30	21.34 ± 3.73	0.98(0.93,1.04)	0.487		
Agreeableness	24.61 ± 4.03	23.81 ± 4.28	0.95(0.91,0.99)	0.040		
Conscientiousness	24.65 ± 4.16	24.07 ± 4.26	0.97(0.93,1.01)	0.143		
Neuroticism	14.23 ± 3.96	15.04 ± 3.62	1.05(1.01,1.10)	0.027	1.05(1.01,1.10)	0.027
Openness	20.11 ± 4.29	20.36 ± 4.10	1.01(0.97,1.06)	0.518		

## Discussion

The findings of this study provide valuable insights into the characteristics, behaviors, and risk factors associated with truck driver crashes, shedding light on important issues related to road safety and crash prevention. The study revealed that a significant proportion of truck drivers have experienced crashes in the last three years. In addition, fatigue-related crashes, sleep-related crashes, and crashes resulting in fatality are common among them.

The findings of this study unveiled that drug use, texting while driving, not wearing seat belts, accumulating driving fines, using sleeping pills, sleep driving, longer driving hours before rest, experiencing fatigue while driving, high visual demands, a careless driving style, and higher levels of neuroticism all contribute to an increased risk of crashes among truck drivers.

Alarmingly, over a quarter of the drivers (28.4%) reported being involved in at least one crash within the last three years, with 12.1% reporting one crash, and 10% noting two crashes. This statistic becomes even more concerning when considering that 6.3% of drivers experienced three or more crashes during this timeframe. These findings underscore the high-risk nature of the profession and the pressing need for targeted safety measures. This result contrasts with a previous study conducted in the US among commercial motor vehicle drivers, where a higher percentage of drivers, specifically 38.6%, experienced at least one crash in their lifetime, and 16.6% reported two or more crashes [46]. Another study on long-haul truck drivers indicated that 35% of drivers reported at least one crash and 12% reported two or more in their career as a truck driver [13]. The current study focuses on a more recent timeframe (the last 3 years) and may have a narrower scope in terms of crash reporting compared to the previous studies, which covered crashes over a driver’s entire lifetime or career period.

Our findings revealed that the majority of crashes (42.5%) occurred between midnight and 6:00 a.m., emphasizing the significant role of circadian rhythms and driving duration in fatigue levels and driving performance [47]. The findings revealed a concerning prevalence of fatigue and sleep-related crashes among the participants. Over their lifetime, nearly a quarter (24.2%) reported experiencing at least one sleep-related crash, while 40.5% had experienced at least one fatigue-related crash. These figures underscore the critical role of driver fatigue and sleep deprivation in traffic incidents. A survey conducted among drivers in Saudi Arabia revealed that 11.6% of crashes within the past six months were sleep-related [48]. The current study highlights a significant relationship between driving time and the likelihood of fatigue-related crashes. Drivers involved in fatigue-related crashes had significantly longer driving times before the crash compared to those in non-fatigue-related crashes. This finding underscores the impact of prolonged driving on driver fatigue and its potential role in increasing crash risk. Evidence indicates that truck drivers identify prolonged driving time as the most significant factor contributing to fatigue [49]. The temporal pattern of crashes in this study further underscores the role of fatigue in crash causation. A higher proportion of crashes occurring between midnight and 6:00 a.m. (83%) were fatigue-related compared to those between 7:00 a.m. and midnight (61.5%). The odds of a crash being fatigue-related were three times higher during the early morning hours. Consistent with these findings, a previous study reported that fatigue driving is frequently observed during midnight to dawn (OR =  2.72) [50].Regarding the characteristics and behaviors of individuals, the study found that both drug use and the use of mobile devices for sending or reading text messages were notable predictors of crashes. Specifically, the odds of a crash were almost 3 fold higher for individuals involved in sending/reading messages compared to making/answering phone calls. A systematic review and meta-analysis of naturalistic driving studies found that tasks like texting, which divert drivers’ attention from the road, pose a higher risk of safety-critical events compared to tasks like talking on the phone, which allow drivers to keep their eyes on the road [51]. Another study, which used naturalistic driving study data and propensity score weighting approaches, found that visual-manual tasks involving cellphones had consistently higher odds ratios (ORs) for crash risk (ranging from 3.47 to 6.63) compared to overall cellphone distraction and cellphone talking (with ORs ranging from 0.63 to 4.15) [52]. Typing and reading text messages while driving significantly impairs drivers’ ability to focus on the road, respond to critical traffic events, stay within their lane, and maintain a consistent speed and following distance [53].

In the present study, the likelihood of being involved in a crash was twice as high for individuals who used drugs. A systematic review and meta-analysis that investigated the risk of RTCs caused by eleven different drugs found that the use of drugs was associated with increases in crash risk [54]. Various drugs have been found to impair driving skills, affecting tasks such as vigilance, reaction time, attention, and vehicle control. Different drugs or combinations have unique impairing effects that can increase crash risk by encouraging risk-taking, diminishing visual scanning, impairing judgment, or causing inattention [55].

In terms of vehicle and driving characteristics, absence of seatbelt use and driving fines were important predictors of crashes. Notably, the odds of a crash were 18 times higher for drivers who had more than 6 driving fines in the last year. Evidence showed that in commercial vehicle drivers, driving violations had negative effects on crash involvement [56]. Investigation of violations in professional truck drivers showed that an increase of one unit in both ordinary and aggressive violations elevated the likelihood of experiencing a traffic crash in the last year by 37% and 42%, respectively [57].

The current study implies that not wearing a seatbelt is associated with a twofold increase in the odds of being involved in crashes. A study involving bus and truck drivers found that not using seat belts increases the likelihood of a crash by 2.7 times [58]. It is important to emphasize that wearing seatbelts remains a fundamental safety practice that significantly reduces the risk of severe crashes in commercially licensed drivers [59]. Unbelted drivers face 8.3 times the risk of a fatal crash and 5.2 times the risk of a serious injury crash compared to belted drivers. These disparities in crash risk are influenced by other common risk factors like drunk driving and speeding [60]. Evidence showed that as the vulnerability of the driver community increased, the risk of not wearing a seat belt increased [61]. Furthermore, individuals with high perceived fatigue, excessive daytime sleepiness, and poor mental health status tend to have higher traffic crash risk index scores and are more likely to fail to use seat belts [30].

Among work patterns and sleep-related factors, the study identified several factors associated with an increased risk of crashes among truck drivers. Notably, taking sleeping pills, sleep driving, longer driving hours before rest, and experiencing fatigue while driving were associated with higher odds of being involved in crashes in the last 3 years. These findings emphasize the critical role that sleep-related factors and extended work hours play in crash risk among truck drivers.

Prior research indicated that driver fatigue was a prevalent issue among professional drivers, with those reporting frequent fatigue experiences being more susceptible to crashes. Among the factors contributing to fatigue, extended driving periods stood out as the most significant. This was often driven by an optimism bias, where professional drivers believed that fatigue posed a greater risk to others than themselves and that they could effectively counteract its impact on their driving performance [49]. Crashes involving fatigued commercial vehicle drivers were more common on roadways located over 20 miles away from rest areas or truck stops [62]. A prior study indicated that increased fatigue levels are associated with drivers maintaining shorter time headways when following other vehicles and opting for shorter time headways when changing lanes. Additionally, greater variability in car-following performance was observed as fatigue levels increased [63].

Evidence indicated that individuals with a sleep disorder have a 29% higher likelihood of experiencing inattentiveness while driving when compared to those without a sleep disorder [64]. The use of benzodiazepines has increased in recent years and is a threat to driving safety [65]. Evidence indicates that following either 6 or 7 hours of prior sleep, there is a slight but noticeable level of driving impairment compared to when individuals have had ≥  8 hours of prior sleep. The likelihood of a crash appears to be approximately 30% higher after 6 or 7 hours of prior sleep compared to well-rested individuals [66]. Evidence indicated that while drivers consider sleepy driving a risky behavior, it is not perceived as risky as driving at high speeds [67]. Analyzing the impact of driver sleepiness on driving behavior reveals that when drivers are drowsy, there is a decline in performance in lane-keeping and speed-keeping. Additionally, there is an increase in the proportion of time with eyes closed when drivers are in a sleepy state [68].

The duration of driving tasks and circadian effects significantly increase the likelihood of “near misses” and accidents, impacting driving performance negatively [69]. An analysis of driving and working hours for truck drivers showed that taking breaks from driving was effective in reducing safety-critical events and counteracting the negative effects of prolonged time-on-task [70]. A survey on the influence of sleep need and time-on-task on driver fatigue indicated that both factors negatively impact the driver’s state. Furthermore, it showed that time-on-task can impair driver performance even when there isn’t a heightened sleep need [71]. A study on truck drivers revealed that increasing total rest-break duration consistently reduces fatigue-related crash risk, with two rest breaks generally sufficient for a 10-hour trip. Rest breaks of around 30 minutes are adequate, and taking breaks too soon after a trip begins may reduce their effectiveness [32]. Another study indicated that incorporating one, two, or three rest breaks significantly reduces accident risk by 68%, 83%, and 85%, respectively, compared to drivers who don’t take any breaks [33]. In addition, for truck drivers, extending the rest period between shifts significantly increases sleep duration and moderately enhances driver alertness and performance [72].

This study revealed that for every unit increase in visual demand, there was a corresponding 23% increase in the odds of being involved in a crash. The evidence indicates that combining a visual detection task with driving is challenging [73]. Increased visual demand results in reduced driving speed and greater variability in lane-keeping [74].

In the current study, being careful emerged as the most significant driving style factor associated with reduced crash risk, highlighting the protective nature of careful driving behaviors. A study that investigated the effect of driving style on the accidents of Iranian intercity bus drivers found that drivers with a crash history had a lower score in careful driving style than drivers without a crash history [75]. In another study, unsafe Chinese drivers were also characterized by lower scores in the careful driving style [76].

This study underscored the importance of neuroticism as the most influential personality trait associated with crash risk. Individuals with higher neuroticism scores faced an elevated risk of crashes, with a 5% increase in crash odds for each unit increase in neuroticism score. Investigation of personality traits in a sample of Iranian drivers revealed that neuroticism was associated with a higher reported crash history [77]. Previous studies found that higher neuroticism scores were associated with aggressive violations [78], distracted driving behavior [79], and lower perceptions of negative social outcomes related to speeding [80].

## Conclusion

The study sheds light on the significant prevalence of RTCs among truck drivers. The findings underscore a constellation of factors amplifying crash risks within this occupational group. These outcomes emphasize the multifaceted nature of road safety issues within the trucking industry, indicating the need for targeted interventions and preventive measures to enhance driver safety and reduce the incidence of RTCs among truck drivers.
